# Echocardiographic characterization and markers of cardiovascular risk in adults with sickle cell disease in a Colombian tertiary referral centre: A cross-sectional study

**DOI:** 10.1371/journal.pone.0348383

**Published:** 2026-06-16

**Authors:** Martin E. Arrieta-Mendoza, Juan R. Betancourt, Sebastián Ayala-Zapata, Stephany Barbosa-Balaguera, Christian D. Messu-Llanos, Juan P. Rosales-Melo, Darío F. Andrade-Hoyos, Álvaro Herrera-Escandón, Oswaldo E. Aguilar-Molina

**Affiliations:** 1 Department of Internal Medicine, Faculty of Health, Universidad del Valle, Cali, Colombia; 2 Department of Cardiology, Hospital Universitario del Valle “Evaristo García” E.S.E., Cali, Colombia; 3 Cardiology Residency Programme, Department of Internal Medicine, Faculty of Health, Universidad del Valle, Cali, Colombia; 4 Clínica Colsanitas S.A., Clínica Sebastián de Belalcázar, Servicio de Cardiología, Cali, Colombia; 5 School of Medicine, Faculty of Health, Universidad del Valle, Cali, Colombia; Université Claude Bernard Lyon 1, FRANCE

## Abstract

Sickle cell disease (SCD) is associated with substantial cardiovascular morbidity, but echocardiographic data from Latin American populations remain scarce. We aimed to characterise the structural, functional, and haemodynamic echocardiographic profile of adults with SCD attending a tertiary referral centre in Cali, Colombia. We conducted an observational, cross-sectional study based on systematic review of medical records and transthoracic echocardiography reports of consecutive adult patients (≥18 years) with confirmed SCD evaluated between January 2022 and December 2024. Patients with complex congenital heart disease, severe valvular disease of unrelated aetiology, pregnancy, or echocardiograms of insufficient quality were excluded. Of 669 patients screened, 57 met inclusion criteria. Reporting followed STROBE recommendations. The median age was 24 years (interquartile range [IQR] 21–32) and 59.6% were female; the SS genotype was the most frequent (76.4%) and 68.5% were on hydroxyurea. Median haemoglobin was 8.5 g/dL (IQR 7.1–10.0) and median NT-proBNP 491 pg/mL (IQR 98–1290). Most patients had preserved left ventricular dimensions and systolic function (median ejection fraction 63%, IQR 57–66.5; mean global longitudinal strain −18.9% ± 2.9). Right ventricular function was preserved (mean tricuspid annular plane systolic excursion 25.4 ± 4.6 mm). Left ventricular geometry was normal in 42.1%, with concentric remodelling in 10.5%, concentric hypertrophy in 21.1%, and eccentric hypertrophy in 26.3%. Diastolic function was normal in 71.4%. Valvular disease, when present, was predominantly mild. Tricuspid regurgitation velocity exceeded 2.5 m/s in 14 of 36 patients with measurable TRV (38.9%; 24.6% of the whole cohort) and exceeded 3.0 m/s in 6 patients (16.7% of those with measurable TRV), identifying a substantial subgroup at intermediate-to-high probability of pulmonary hypertension. In this Colombian cohort of relatively young adults with SCD, cardiac structure and biventricular function were largely preserved, but nearly one-third of patients had echocardiographic findings suggestive of pulmonary hypertension. These findings support the routine use of transthoracic echocardiography as an accessible tool for early cardiovascular risk stratification in adults with SCD in low- and middle-income settings.

## Introduction

Sickle cell disease (SCD) is an inherited haemoglobinopathy caused by a single point mutation in the HBB gene that produces an abnormal β-globin chain. The resulting haemoglobin S polymerises under hypoxic conditions, deforming erythrocytes and triggering chronic haemolysis, recurrent vaso-occlusion, and progressive multi-organ damage [1,2]. Globally, more than 7.7 million people are estimated to live with SCD, with the highest burden concentrated in sub-Saharan Africa and Latin America [3]. In Colombia, the national prevalence is approximately 0.33 cases per 100,000 inhabitants, but rises to 0.66 per 100,000 in Valle del Cauca, where Afro-descendant ancestry is highly represented [4].

Improvements in early diagnosis, infection prophylaxis, and disease-modifying therapies—particularly hydroxyurea—have substantially extended survival in SCD over the last three decades. As a consequence, cardiopulmonary complications have emerged as the leading cause of premature death in adults with SCD, accounting for 26–43% of mortality in published cohorts [5,6]. Reported manifestations include left ventricular hypertrophy and chamber dilatation, diastolic dysfunction, pulmonary hypertension, valvular regurgitation, atrial arrhythmias, and—less commonly—overt systolic dysfunction [7–9]. Tricuspid regurgitation velocity (TRV) >2.5 m/s, an E/e′ ratio >10, and impaired global longitudinal strain (GLS) have all been associated with increased mortality in this population [8,10]. Transthoracic echocardiography (TTE) remains the cornerstone non-invasive imaging modality for cardiovascular assessment in SCD: it is widely available, inexpensive, free of ionising radiation, and capable of detecting subclinical structural and functional alterations long before symptoms develop [9,11].

Despite the relevance of cardiovascular involvement in SCD, echocardiographic data from Latin American populations are very limited. Most published cohorts originate from North America, Europe, the Middle East, or sub-Saharan Africa [8,11–14], and direct extrapolation to populations with different genetic background, age structure, comorbidity profile, and access to disease-modifying therapy is questionable. To our knowledge, no contemporary cohort has systematically described the echocardiographic profile of adults with SCD in the Colombian Pacific—a region with one of the highest concentrations of Afro-descendant population in the country.

The aim of this study was to characterise the clinical and echocardiographic features of adults with SCD attending a tertiary referral centre in Cali, Colombia, with particular focus on structural, systolic, diastolic, valvular, and haemodynamic parameters relevant to cardiovascular risk stratification.

## Methods

### Study design and reporting

We conducted an observational, descriptive, cross-sectional study based on systematic review of electronic medical records and TTE reports of consecutive adult patients with a confirmed diagnosis of SCD evaluated at the Hospital Universitario del Valle “Evaristo García” E.S.E. (HUV), a tertiary referral centre in Cali, Colombia, between January 2022 and December 2024. Reporting follows the Strengthening the Reporting of Observational Studies in Epidemiology (STROBE) recommendations [15]; a completed STROBE checklist is provided as Supporting Information (S1 Checklist).

### Setting and participants

The HUV is the largest public tertiary hospital in southwestern Colombia and serves as the regional referral centre for haemoglobinopathies in Valle del Cauca and adjacent departments of the Pacific region.

**Inclusion criteria:** (i) age ≥ 18 years; (ii) confirmed diagnosis of SCD established by haemoglobin electrophoresis or DNA-based methods; and (iii) at least one complete TTE performed and reported during the study period.

**Exclusion criteria:** (i) complex structural congenital heart disease unrelated to SCD; (ii) severe valvular heart disease of aetiology unrelated to SCD; (iii) incomplete TTE studies or studies of insufficient image quality for analysis; (iv) inability to verify clinical or genetic confirmation of SCD; and (v) pregnancy at the time of TTE.

### Data collection and definitions

Demographic, clinical, laboratory, and echocardiographic variables were extracted using a standardised electronic data collection form with double data entry to minimise transcription errors. The authors accessed the electronic medical records and transthoracic echocardiography reports of the Hospital Universitario del Valle for research purposes between 20/11/2025 and 28/02/2026, after the approval of the Research Ethics Committee. During data collection, the authors had access to information that could identify individual participants; all data were de-identified immediately after extraction and before analysis. The date of the most recent blood transfusion was not consistently recorded in the source electronic medical records and could therefore not be reliably ascertained for all patients; this limitation is discussed below. Echocardiographic measurements were obtained from validated reports issued by board-certified cardiologists with level III training in echocardiography, performed on commercially available ultrasound systems and reported in accordance with the recommendations of the American Society of Echocardiography (ASE) and the European Association of Cardiovascular Imaging (EACVI) [16].

Left ventricular geometry was classified using relative wall thickness and indexed left ventricular mass per ASE/EACVI criteria [16]. Diastolic function was graded using the 2016 ASE/EACVI algorithm [17]. The probability of pulmonary hypertension was assessed using two complementary frameworks. First, the 2022 European Society of Cardiology/European Respiratory Society (ESC/ERS) echocardiographic criteria, in which TRV > 2.5 m/s and >2.8 m/s define intermediate and high probability of pulmonary hypertension, respectively, in the absence of other echocardiographic signs [18]. Second, the SCD-specific threshold of TRV > 3.0 m/s, which has been validated in the SCD population as more specifically associated with pre-capillary pulmonary hypertension confirmed by right heart catheterisation, and which we therefore report alongside the ESC/ERS thresholds [8,21]. Global longitudinal strain (GLS) was measured by two-dimensional speckle-tracking echocardiography in vendor-specific software and is reported as the absolute mean value with standard deviation (SD).

### Statistical analysis

Continuous variables were tested for normality using the Shapiro–Wilk test and visual inspection of histograms; they are reported as mean ± SD when normally distributed and as median with interquartile range (IQR) otherwise. Categorical variables are reported as absolute frequencies and percentages. The analysis was descriptive, in line with the cross-sectional design and pre-defined objectives. Missing data were not imputed and the denominator is reported for each variable. Statistical analyses were performed using Stata version 18 (StataCorp LLC, College Station, TX, USA).

### Ethics

The study protocol was reviewed and approved by the Research Ethics Committee of the Hospital Universitario del Valle “Evaristo García” E.S.E. (Cali, Colombia) on 13/11/2025 (Minute No. 21; project code INT352; internal code 068–2025), and was classified as risk-free research in accordance with Resolution 8430 of 1993 of the Colombian Ministry of Health. Given the retrospective nature of the study and the use of fully anonymised data, the requirement for individual informed consent was waived by the same Committee. All procedures were conducted in accordance with the Declaration of Helsinki. Patient data were de-identified before extraction and analysis.

## Results

### Study population

**Fig 1:**
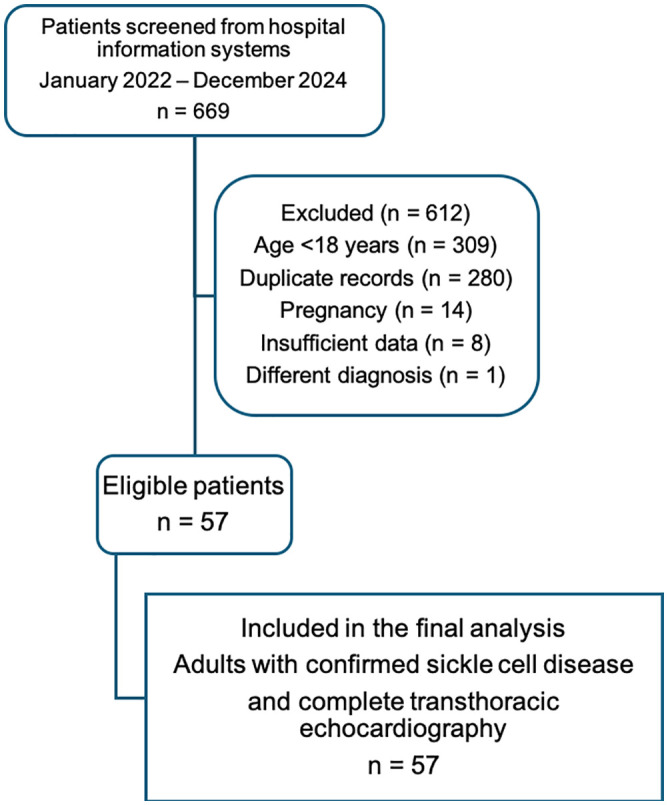
Study flow diagram. Of 669 patients screened from hospital information systems between January 2022 and December 2024, 612 were excluded (309 aged <18 years, 280 duplicate records, 14 pregnant, 8 with insufficient data, 1 with a different diagnosis), leaving 57 adults with confirmed sickle cell disease and complete transthoracic echocardiography for the final analysis.

Of 669 adult records initially screened from the hospital information systems, 612 were excluded according to the criteria detailed in **Fig 1**, leaving 57 patients in the final analysis.

**Fig 2:**
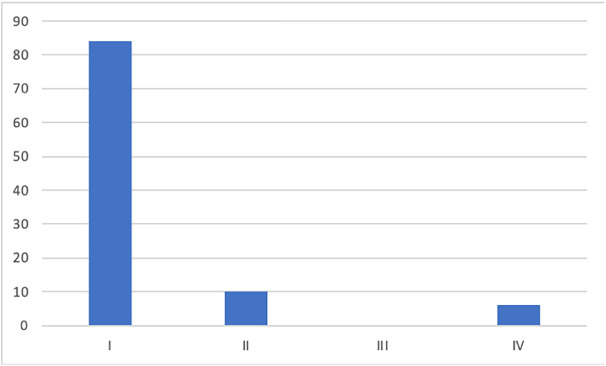
Distribution of New York Heart Association (NYHA) functional class. Bar chart showing the proportion of patients in each NYHA class. NYHA: I, no limitation of physical activity; II, slight limitation; III, marked limitation; IV, symptoms at rest.

Baseline clinical and laboratory characteristics are summarised in **Table 1**. The median age was 24 years (IQR 21–32), and 34 patients (59.6%) were female. The SS genotype was the most frequent, present in 42 of 55 patients with known genotype (76.4%); SC, Sβ + , Sβ0, and other genotypes accounted for the remainder. A vaso-occlusive crisis within the previous 30 days was reported in 37 patients (66.1%), and 37 of 54 patients with available data (68.5%) were receiving hydroxyurea at the time of evaluation. Median haemoglobin was 8.5 g/dL (IQR 7.1–10.0); median creatinine 0.6 mg/dL (IQR 0.5–0.7); median NT-proBNP 491 pg/mL (IQR 98–1290; n = 17). New York Heart Association (NYHA) functional class distribution is shown in **Fig 2**: 84% of patients were in class I, 10% in class II, and 6% in class IV. The date of the most recent blood transfusion could not be ascertained reliably for all patients, as it was not consistently recorded in the source electronic medical records (see Methods and Limitations); the proportion of patients transfused within the three months preceding echocardiography therefore could not be calculated. This is acknowledged as a limitation that may have influenced several biological and echocardiographic parameters, particularly haemoglobin, NT-proBNP, and TRV.

**Table 1:** Baseline clinical and laboratory characteristics of the study population.

Variable	Total (n = 57)
Demographics	
Age, years, median (IQR)	24 (21–32)
Female sex, n (%)	34 (59.6)
Body mass index, kg/m², median (IQR)	22.0 (19.7–25.1)
Body surface area, m², median (IQR)	1.7 (1.6–1.7)
Sickle cell disease genotype, n (%)	
SS (homozygous)	42 (76.4)
SC	6 (10.9)
Sβ⁺	4 (7.3)
Sβ⁰	2 (3.6)
Other	1 (1.8)
Clinical and laboratory variables	
Vaso-occlusive crisis within 30 days, n (%)	37 (66.1)
Haemoglobin, g/dL, median (IQR)	8.5 (7.1–10.0)
Creatinine, mg/dL, median (IQR)	0.6 (0.5–0.7)
NT-proBNP, pg/mL, median (IQR)	491 (98–1290)
Hydroxyurea use, n (%)	37 (68.5)

IQR, interquartile range; NT-proBNP, N-terminal pro–B-type natriuretic peptide; SC, heterozygous compound HbS/HbC; Sβ ⁺ /Sβ⁰, sickle β-thalassaemia; SS, homozygous HbS.

### Structural and systolic function

Echocardiographic measurements are summarised in **Table 2**. Left ventricular dimensions, wall thickness, and indexed mass were within normal reference ranges in most patients. Left ventricular geometry was normal in 24 patients (42.1%); concentric remodelling was observed in 6 (10.5%), concentric hypertrophy in 12 (21.1%), and eccentric hypertrophy in 15 (26.3%). Median left ventricular ejection fraction was 63% (IQR 57.0–66.5), and mean GLS was −18.9% ± 2.9 (measured in 8 patients), both within reference ranges. Right ventricular systolic function was preserved (mean tricuspid annular plane systolic excursion 25.4 mm ± 4.6; median tricuspid annulus systolic velocity [S′] 15 cm/s, IQR 13–16).

**Table 2:** Echocardiographic measurements of the study population (n = 57).

Parameter	Value
Left ventricular structure	
LV end-diastolic diameter, mm, median (IQR)	49 (46–53)
LV end-systolic diameter, mm, median (IQR)	32 (28–35)
Septal wall thickness, mm, median (IQR)	9 (8–11)
Posterior wall thickness, mm, median (IQR)	9 (8–10)
Relative wall thickness, median (IQR)	0.4 (0.3–0.4)
LV mass index, g/m², median (IQR)	93.4 (74.9–116.7)
LV end-systolic volume, mL, median (IQR)	40 (34.8–54.5)
LV end-diastolic volume, mL, median (IQR)	110.5 (96.8–134.0)
Indexed LV end-diastolic volume, mL/m², median (IQR)	66.4 (56.3–81.1)
Indexed LV end-systolic volume, mL/m², median (IQR)	24.6 (20.6–32.2)
Stroke volume, mL, median (IQR)	65 (56.5–81.5)
Indexed left atrial volume, mL/m², median (IQR)	36.1 (25.2–42.7)
Indexed right atrial volume, mL/m², median (IQR)	25.5 (18.1–33.0)
LVOT diameter, mm, median (IQR)	21 (19–22)
Aortic root, mm, median (IQR)	31 (28–32)
Systolic function	
LV ejection fraction, %, median (IQR)	63 (57.0–66.5)
Global longitudinal strain, %, mean ± SD	−18.9 ± 2.9
TAPSE, mm, mean ± SD	25.4 ± 4.6
Tricuspid annular S′, cm/s, median (IQR)	15 (13–16)
Diastolic function	
E wave, m/s, mean ± SD	0.9 ± 0.2
E/A ratio, median (IQR)	1.6 (1.3–1.8)
Average E/e′ ratio, mean ± SD	8.1 ± 2.1
Pulmonary haemodynamics	
Tricuspid regurgitation peak velocity, m/s, median (IQR)	2.4 (2.3–2.7)
Estimated pulmonary artery systolic pressure, mmHg, median (IQR)	26.7 (24.2–32.2)
Inferior vena cava diameter, mm, median (IQR)	15 (13–18)
Pulmonary acceleration time, ms, mean ± SD	129 ± 33

IQR, interquartile range; LV, left ventricular; LVOT, left ventricular outflow tract; SD, standard deviation; TAPSE, tricuspid annular plane systolic excursion.

### Valvular and diastolic function

**Fig 3:**
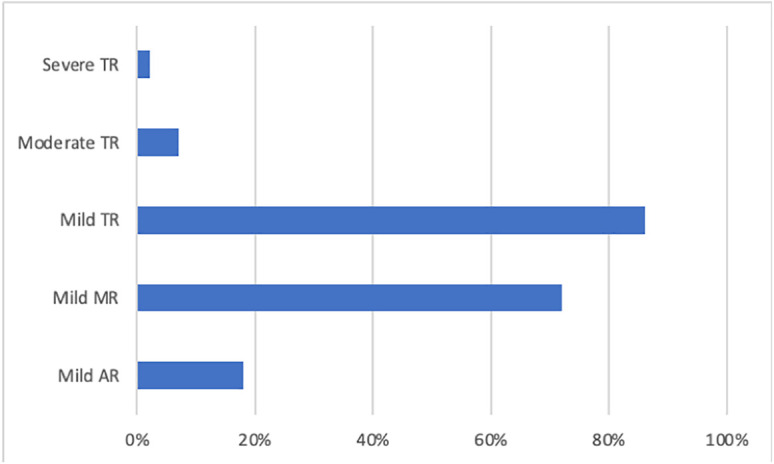
Prevalence of valvular regurgitation. Horizontal bar chart showing the proportion of patients with mild, moderate, or severe regurgitation of the aortic, mitral, and tricuspid valves. AR, aortic regurgitation; MR, mitral regurgitation; TR, tricuspid regurgitation.

Valvular abnormalities, when present, were predominantly mild (**Fig 3**). Mild tricuspid regurgitation was the most frequent finding (≈86%), followed by mild mitral regurgitation (≈72%) and mild aortic regurgitation (≈18%). Moderate tricuspid regurgitation was observed in approximately 7% of patients and severe tricuspid regurgitation in approximately 2%.

**Fig 4:**
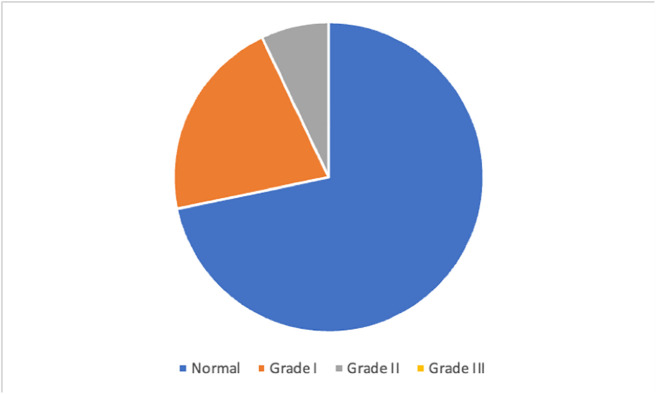
Distribution of diastolic function. Pie chart showing the proportion of patients with normal diastolic function and with grades I, II, and III diastolic dysfunction.

Diastolic function was normal in 41 patients (71.4%); grade I (impaired relaxation) was present in 12 (21.4%) and grade II in 4 (7.1%); no patient had grade III diastolic dysfunction (**Fig 4**). The median E/A ratio was 1.6 (IQR 1.3–1.8), and the mean E/e′ ratio was 8.1 ± 2.1. The median indexed left atrial volume was 36.1 mL/m² (IQR 25.2–42.7; n = 54), at the upper limit of normal, and the median indexed right atrial volume was 25.5 mL/m² (IQR 18.1–33.0; n = 51), within normal limits.

### Pulmonary haemodynamics

TRV was measurable in 36 of the 57 patients (63.2%); in the remaining 21 patients no adequate tricuspid regurgitant jet was available. Among patients with measurable TRV, the median TRV was 2.4 m/s (IQR 2.3–2.7). Fourteen patients had a TRV > 2.5 m/s, corresponding to 38.9% of those with measurable TRV (14 of 36) and 24.6% of the whole cohort (14 of 57); this represents an intermediate echocardiographic probability of pulmonary hypertension according to the 2022 ESC/ERS criteria [18]; of these, 6 patients had a TRV > 3.0 m/s (16.7% of those with measurable TRV; 10.5% of the whole cohort), with the remainder falling between 2.5 and 3.0 m/s — a range that encompasses the ESC/ERS high-probability threshold of >2.8 m/s [18] and partially overlaps with the SCD-specific threshold of >3.0 m/s most consistently associated with pre-capillary pulmonary hypertension confirmed by right heart catheterisation [8,21]. The median estimated pulmonary artery systolic pressure was 26.7 mmHg (IQR 24.2–32.2; n = 36), the median inferior vena cava diameter was 15 mm (IQR 13–18), and the mean pulmonary acceleration time was 129 ± 33 ms.

### Exploratory comparison according to tricuspid regurgitation velocity

As suggested during peer review, we performed a descriptive exploratory comparison between patients with measurable TRV > 2.5 m/s (n = 14) and those with measurable TRV ≤ 2.5 m/s (n = 22). The two subgroups were similar in age (median 24 vs 26 years), haemoglobin concentration (median 8.5 vs 8.4 g/dL), left ventricular ejection fraction (median 63.0% vs 60.3%), left ventricular mass index (median 99.5 vs 99.8 g/m²) and average E/e′ ratio (median 8.6 vs 7.6). As expected by design, estimated pulmonary artery systolic pressure was substantially higher in the elevated-TRV group (median 34.8 vs 24.2 mmHg). Indexed left and right atrial volumes tended to be larger in patients with TRV > 2.5 m/s (median indexed left atrial volume 38.4 vs 33.2 mL/m²; indexed right atrial volume 30.4 vs 22.0 mL/m²), and TAPSE was slightly lower (median 23.5 vs 26.0 mm). These exploratory observations should be interpreted with caution given the very small numbers and the descriptive nature of the analysis; formal hypothesis testing and multivariable modelling were not pre-specified and are deferred to our planned prospective multicentre follow-up study.

## Discussion

In this contemporary cross-sectional study of 57 adults with SCD attending a tertiary referral centre in Cali, Colombia, we found that cardiac structure and biventricular systolic function were largely preserved at the population level, while approximately one-third of patients had echocardiographic findings consistent with an intermediate-to-high probability of pulmonary hypertension. To our knowledge, this is the first systematic echocardiographic characterisation of adults with SCD in the Colombian Pacific, a region with one of the highest concentrations of Afro-descendant population in Latin America.

Our cohort was younger (median age 24 years) than most published series, in which median ages typically range between 32 and 37 years [8,11,19]. Two factors may explain this difference. First, our centre acts as a regional referral hub for both paediatric and adult haemoglobinopathy care, with continuous transition of younger patients into adult clinics. Second, premature mortality in SCD remains high in low- and middle-income settings, which truncates the age distribution of prevalent adult cases [3,5]. This younger age structure has direct implications for the interpretation of our findings, as both chamber remodelling and pulmonary hypertension increase with cumulative disease exposure [8,11].

The female predominance (59.6%) in our sample is comparable to that reported by Parent et al. [8] and Naoman et al. [13]. Because SCD is an autosomal recessive disorder, equal sex distribution is biologically expected; the observed predominance is most likely explained by greater contact of women with health services and referral patterns rather than a true epidemiological difference.

Ventricular and atrial volumes—both absolute and indexed to body surface area—were within normal reference limits in the majority of patients, in contrast with the cohort reported by Abdul-Mohsen [11], in which up to one-quarter of patients had chamber dilatation, and more consistent with younger Western series [12,19]. Indexed left ventricular mass was likewise preserved. Two factors plausibly account for these results: the relatively young age of our cohort (with a shorter cumulative duration of chronic anaemia and volume overload) and the high proportion of patients on hydroxyurea (68.5%), which has been shown to reduce haemolysis and may attenuate adverse cardiac remodelling [20].

Left ventricular systolic function was preserved at the population level (median ejection fraction 63%; mean GLS −18.9%), in agreement with previous series [11,12,16,19]. The mean GLS value falls within the normal range reported for healthy adults of similar age and is comparable to data from European cohorts of patients with SCD [12]. Right ventricular longitudinal function was also preserved, in contrast with the report by Chiadika et al. [14] in which approximately one-third of patients had abnormal tricuspid annular plane systolic excursion. Differences in age structure and disease burden are the most likely explanation.

Diastolic function was normal in most patients (71.4%), and no patient had grade III dysfunction. This contrasts with cohorts in which the majority of patients showed some degree of diastolic dysfunction [17] but is consistent with the Saudi cohort reported by Abdul-Mohsen [11], in which dysfunction—when present—was predominantly mild. Diastolic dysfunction has been independently associated with mortality in SCD [19]; the relatively low prevalence in our cohort may reflect both younger age and high hydroxyurea use, but mortality outcomes could not be evaluated in this cross-sectional design.

The most clinically relevant finding of our study is that 14 of 36 patients with measurable TRV (38.9%) had a TRV > 2.5 m/s and 6 (16.7%) had a TRV > 3.0 m/s. Two complementary thresholds are commonly applied in this context. Under the 2022 ESC/ERS general framework, TRV > 2.5 m/s and >2.8 m/s correspond to intermediate and high echocardiographic probability of pulmonary hypertension, respectively, in the absence of additional echocardiographic signs [18]. In the SCD-specific literature, TRV ≥ 2.5 m/s has been independently associated with increased mortality risk [21], while TRV ≥ 3.0 m/s has the highest positive predictive value for pre-capillary pulmonary hypertension confirmed by right heart catheterisation, with values below this threshold yielding a substantial proportion of false-positive results [8]. The proportion of patients with TRV > 2.5 m/s in our cohort is remarkably consistent with the landmark French cohort reported by Parent et al. [8] (approximately 27%) and with North American series [22,23], suggesting that the burden of pulmonary hypertension risk in our Colombian cohort is comparable to that reported elsewhere despite the younger median age. TRV > 2.5 m/s has been shown to confer a more than ten-fold increase in mortality risk in SCD when combined with other markers such as elevated E/e′ ratio [10]; identifying these patients echocardiographically may therefore guide earlier referral for confirmatory right heart catheterisation and intensification of disease-modifying therapy [9].

From a public-health perspective, our data support the routine use of TTE as an accessible, non-invasive tool for cardiovascular risk stratification in adults with SCD in low- and middle-income settings, where access to advanced cardiovascular imaging modalities is often limited. Identification of an intermediate-to-high probability of pulmonary hypertension on TTE should prompt further evaluation and consideration of early intensification of disease-modifying therapy, rather than waiting for symptomatic deterioration.

### Strengths and limitations

**Strengths.** This is, to our knowledge, the first systematic echocardiographic characterisation of adults with SCD in the Colombian Pacific. All echocardiograms were performed and reported by board-certified cardiologists with level III training using a standardised institutional protocol, and we report a comprehensive set of structural, systolic, diastolic, valvular, and haemodynamic parameters, including GLS by speckle-tracking echocardiography.

**Limitations.** Our study has several limitations that must be acknowledged. First, the retrospective single-centre design limits external generalisability and precludes causal inference. Second, the absence of a healthy local control group prevents direct comparison of echocardiographic parameters with reference values derived from the same population. Third, the modest sample size (n = 57) and the descriptive nature of the analysis precluded multivariable modelling of predictors of pulmonary hypertension or diastolic dysfunction; in particular, the small number of patients with TRV > 2.5 m/s (n = 14) and especially with TRV > 3.0 m/s (n = 6) precluded a robust comparative subgroup analysis between patients with elevated and normal TRV, which is an important objective for our planned prospective multicentre follow-up study. Fourth, the date and frequency of the most recent blood transfusion were not consistently recorded in the source electronic medical records and could therefore not be ascertained reliably for all patients; because recent transfusion may modify haemoglobin concentration, haemolytic markers, NT-proBNP, and TRV, this represents an additional source of potential confounding that will be addressed prospectively in future work. Fifth, mortality and longitudinal cardiovascular outcomes were not evaluated. Sixth, right heart catheterisation was not systematically performed; pulmonary hypertension probability is therefore based on echocardiographic criteria alone. Finally, the relatively young age of the cohort may have underestimated the burden of cardiovascular involvement that typically accumulates beyond the fifth decade of life. These limitations should be addressed in future prospective multicentre studies including longitudinal follow-up, systematic recording of transfusion history, comparative subgroup analyses, and right heart catheterisation in patients with elevated TRV.

## Conclusion

In this Colombian cohort of relatively young adults with SCD, cardiac structure and biventricular systolic and diastolic function were largely preserved. Nonetheless, nearly one-third of patients had echocardiographic findings consistent with an intermediate-to-high probability of pulmonary hypertension, identifying a clinically relevant subgroup at increased cardiovascular risk. Our findings support the use of transthoracic echocardiography as a routine, accessible tool for early cardiovascular risk stratification in adults with SCD in Latin American and other low- and middle-income settings, and provide a contemporary local reference against which future prospective and interventional studies can be designed.

## Supporting information

S1 Checklist STROBE checklist for cross-sectional studies. (DOCX)

S2 Dataset De-identified individual-level dataset (n = 57). XLSX file containing four sheets: README, Data, Data dictionary, and Code legends. (XLSX)
