# Supplementary material for: Echocardiographic characterization and markers of cardiovascular risk in adults with sickle cell disease in a Colombian tertiary referral centre: A cross-sectional study
Source: PLoS One. 2026 Jun 16;21(6):e0348383. doi: 10.1371/journal.pone.0348383 (PMC13271432; doi:10.1371/journal.pone.0348383)
Supplement: S1 Checklist — (DOCX) [file pone.0348383.s001.docx]

**S1 Checklist. STROBE Statement—checklist of items that should be included in reports of cross-sectional studies.**

*Manuscript: “Echocardiographic characterization and markers of cardiovascular risk in adults with sickle cell disease in a Colombian tertiary referral centre: a cross-sectional study.” Page numbers refer to the revised manuscript file.*

| **Section/Topic** | **Item No** | **Recommendation** | **Reported in (section / page)** |
| --- | --- | --- | --- |
| **Title and abstract** | | | |
| Title and abstract | 1 | (a) Indicate the study’s design with a commonly used term in the title or the abstract | Title (p. 1); Abstract (p. 2) |
|  |  | (b) Provide in the abstract an informative and balanced summary of what was done and what was found | Abstract (p. 2) |
| **Introduction** | | | |
| Background/rationale | 2 | Explain the scientific background and rationale for the investigation being reported | Introduction (p. 2) |
| Objectives | 3 | State specific objectives, including any prespecified hypotheses | Introduction, final paragraph (pp. 2–3) |
| **Methods** | | | |
| Study design | 4 | Present key elements of study design early in the paper | Methods – “Study design and reporting” (p. 3) |
| Setting | 5 | Describe the setting, locations, and relevant dates, including periods of recruitment, exposure, follow-up, and data collection | Methods – “Study design and reporting” and “Setting and participants”; study period Jan 2022–Dec 2024 (p. 3) |
| Participants | 6 | (a) Give the eligibility criteria, and the sources and methods of selection of participants | Methods – “Setting and participants”; Inclusion and Exclusion criteria (pp. 3–4) |
| Variables | 7 | Clearly define all outcomes, exposures, predictors, potential confounders, and effect modifiers. Give diagnostic criteria, if applicable | Methods – “Data collection and definitions” (p. 4) |
| Data sources/ measurement | 8* | For each variable of interest, give sources of data and details of methods of assessment (measurement). Describe comparability of assessment methods if there is more than one group | Methods – “Data collection and definitions” (p. 4) |
| Bias | 9 | Describe any efforts to address potential sources of bias | Methods – “Data collection and definitions” (standardised electronic form, double data entry, reports by level III–trained cardiologists per ASE/EACVI standards) (p. 4); Limitations (p. 9) |
| Study size | 10 | Explain how the study size was arrived at | Consecutive eligible patients during the study period; no a priori sample-size calculation (descriptive study). Methods (p. 3); flow diagram, Fig 1 (p. 5) |
| Quantitative variables | 11 | Explain how quantitative variables were handled in the analyses. If applicable, describe which groupings were chosen and why | Methods – “Statistical analysis”; categorisation of LV geometry and diastolic function per ASE/EACVI criteria in “Data collection and definitions” (p. 4) |
| Statistical methods | 12 | (a) Describe all statistical methods, including those used to control for confounding | Methods – “Statistical analysis” (p. 4) |
|  |  | (b) Describe any methods used to examine subgroups and interactions | Methods – “Statistical analysis” (descriptive); exploratory TRV subgroup comparison in Results (p. 4; p. 7) |
|  |  | (c) Explain how missing data were addressed | Methods – “Statistical analysis” (no imputation; denominator reported for each variable) (p. 4) |
|  |  | (d) If applicable, describe analytical methods taking account of sampling strategy | Not applicable (no complex sampling design) |
|  |  | (e) Describe any sensitivity analyses | Not applicable (descriptive analysis; none performed) |
| **Results** | | | |
| Participants | 13* | (a) Report numbers of individuals at each stage of study—eg numbers potentially eligible, examined for eligibility, confirmed eligible, included in the study, completing follow-up, and analysed | Results – “Study population”; Fig 1 flow diagram (p. 5) |
|  |  | (b) Give reasons for non-participation at each stage | Fig 1 flow diagram (exclusions detailed) (p. 5) |
|  |  | (c) Consider use of a flow diagram | Fig 1 (p. 5) |
| Descriptive data | 14* | (a) Give characteristics of study participants (eg demographic, clinical, social) and information on exposures and potential confounders | Results – “Study population”; Table 1 (pp. 5, 13) |
|  |  | (b) Indicate number of participants with missing data for each variable of interest | Denominators reported throughout Results and Tables (e.g., genotype n = 55, hydroxyurea n = 54, NT-proBNP n = 17, measurable TRV n = 36) (pp. 5–6) |
| Outcome data | 15* | Report numbers of outcome events or summary measures | Results – “Structural and systolic function”, “Valvular and diastolic function”, “Pulmonary haemodynamics”; Table 2 (pp. 6, 13) |
| Main results | 16 | (a) Give unadjusted estimates and, if applicable, confounder-adjusted estimates and their precision (eg, 95% confidence interval). Make clear which confounders were adjusted for and why they were included | Descriptive estimates (median [IQR], mean ± SD, proportions with denominators); no adjusted estimates (descriptive design). Results and Table 2 (pp. 6, 13) |
|  |  | (b) Report category boundaries when continuous variables were categorized | TRV thresholds (>2.5, >2.8, >3.0 m/s), LV geometry categories and diastolic grades defined in Methods (p. 4) and reported in Results (p. 6) |
|  |  | (c) If relevant, consider translating estimates of relative risk into absolute risk for a meaningful time period | Not applicable (no relative-risk estimates) |
| Other analyses | 17 | Report other analyses done—eg analyses of subgroups and interactions, and sensitivity analyses | Results – “Exploratory comparison according to tricuspid regurgitation velocity” (p. 7) |
| **Discussion** | | | |
| Key results | 18 | Summarise key results with reference to study objectives | Discussion, opening paragraph (p. 7) |
| Limitations | 19 | Discuss limitations of the study, taking into account sources of potential bias or imprecision. Discuss both direction and magnitude of any potential bias | Discussion – “Strengths and limitations” / “Limitations” (p. 9) |
| Interpretation | 20 | Give a cautious overall interpretation of results considering objectives, limitations, multiplicity of analyses, results from similar studies, and other relevant evidence | Discussion (pp. 7–10) |
| Generalisability | 21 | Discuss the generalisability (external validity) of the study results | Discussion (public-health perspective; younger cohort; LMIC setting) and Limitations (pp. 9–10) |
| **Other information** | | | |
| Funding | 22 | Give the source of funding and the role of the funders for the present study and, if applicable, for the original study on which the present article is based | Funding statement provided in the online submission system (PLOS Financial Disclosure), per journal policy |

**Give information separately for exposed and unexposed groups.*

Note: An Explanation and Elaboration article discusses each checklist item and gives methodological background and published examples of transparent reporting. The STROBE checklist is best used in conjunction with this article (freely available on the websites of PLOS Medicine at http://www.plosmedicine.org/, Annals of Internal Medicine at http://www.annals.org/, and Epidemiology at http://www.epidem.com/). Information on the STROBE Initiative is available at http://www.strobe-statement.org.
