# Supplementary material for: Echocardiographic characterization and markers of cardiovascular risk in adults with sickle cell disease in a Colombian tertiary referral centre: A cross-sectional study
Source: PLoS One. 2026 Jun 16;21(6):e0348383. doi: 10.1371/journal.pone.0348383 (PMC13271432; doi:10.1371/journal.pone.0348383)
Supplement: Attachment — Submitted filename: Response_to_Reviewers_4.docx [file pone.0348383.s003.docx]

**Response to Reviewers**

**Manuscript:** PONE-D-26-18282

**Title:** Echocardiographic characterization and markers of cardiovascular risk in adults with sickle cell disease in a Colombian tertiary referral centre: a cross-sectional study

**Journal:** PLOS ONE

**Corresponding author:** Stephany Barbosa-Balaguera

Dear Dr. Connes and Reviewer #1,

We thank you for the constructive evaluation of our manuscript and the opportunity to submit a revised version. We have carefully addressed every point raised by the Academic Editor and Reviewer #1. All changes are visible in the "Revised Manuscript with Track Changes" file. Below we provide a point-by-point response. For clarity, reviewer and editor comments are reproduced verbatim in italics, followed by our response.

**Preliminary note — Data consistency audit and corrections**

Before addressing the specific comments of the Academic Editor and Reviewer #1, we wish to bring an important matter to the Editor’s attention in the interest of full transparency.

In compliance with the PLOS Data Availability policy, we prepared the complete de-identified individual-level dataset (S2 Dataset) to accompany the revised manuscript. While doing so, we conducted a systematic, variable-by-variable consistency check between this dataset and the values reported in Tables 1 and 2 and in the Abstract. This audit identified several discrepancies, which we have corrected. We summarise them here, and all corresponding changes are visible in the marked-up manuscript.

The discrepancies were of three kinds. First, a small number of transcription errors in individual values: median haemoglobin (corrected from 10.2 to 8.5 g/dL), median creatinine (corrected from 0.5 to 0.6 mg/dL), hydroxyurea use (corrected from 40 [71.4%] to 37 of 54 patients with available data [68.5%]), and two left ventricular geometry categories whose percentages had been inadvertently transposed (concentric remodelling, corrected to 10.5%; eccentric hypertrophy, corrected to 26.3%). Additional minor table corrections affected posterior wall thickness, relative wall thickness, and LVOT diameter, which had small rounding inconsistencies with respect to the source dataset. Second, several left ventricular dimensions and volumes in Table 2 required correction to match the dataset (e.g., LV end-diastolic diameter, LV mass index, LV end-diastolic and end-systolic volumes, stroke volume, indexed atrial volumes, and aortic root). We also identified and corrected an error in the Results text concerning indexed atrial volume: the value previously reported as "indexed left atrial volume 25.5 mL/m² (IQR 21.4–30.0), within normal limits" had inadvertently mixed the right atrial value into the left atrial sentence. The revised text now correctly reports the indexed left atrial volume as 36.1 mL/m² (IQR 25.2–42.7; n = 54), at the upper limit of normal, and the indexed right atrial volume as 25.5 mL/m² (IQR 18.1–33.0; n = 51), within normal limits. Third — and most relevant from a methodological standpoint — we identified variables with incomplete data that had been reported without an explicit denominator. Most notably, tricuspid regurgitation velocity (TRV) was measurable in 36 of the 57 patients; the proportion of patients with TRV > 2.5 m/s is now reported transparently both as a fraction of patients with measurable TRV (14 of 36; 38.9%) and of the whole cohort (24.6%), and the 21 patients without a measurable tricuspid regurgitant jet are now explicitly stated. Denominators have likewise been made explicit for NT-proBNP (n = 17) and global longitudinal strain (n = 8).

We emphasise that the study population (n = 57) and all primary analyses are unchanged. These corrections affect reported values and denominators only; they do not alter the study design, the inclusion of patients, or the principal conclusions, which remain fully supported. The revised manuscript now reports the denominator for every variable with incomplete data, consistent with the approach already described in our Statistical analysis section.

All changes are shown in the "Revised Manuscript with Track Changes" file. The accompanying S2 Dataset includes a README sheet listing the available-case denominator for every variable. We are confident that the manuscript and the supporting dataset are now fully concordant and can be independently verified by any reader.

**Response to the Academic Editor’s additional requirements**

**Editor comment 1.** *Please ensure that your manuscript meets PLOS ONE’s style requirements, including those for file naming. The PLOS ONE style templates can be found at the indicated URLs.*

**Response:** We have reviewed both PLOS ONE style templates (main body and title/authors/affiliations) and reformatted the manuscript accordingly. The title page, author list and affiliations, abstract, main body section headings (Introduction, Methods, Results, Discussion, Conclusion, Acknowledgments, Author contributions, Competing interests, Funding, Data availability, References), figure and table legends, and Supporting Information section now follow the PLOS ONE format. Files have been renamed in accordance with PLOS ONE naming conventions: "Manuscript", "Revised Manuscript with Track Changes", "Response to Reviewers", "Cover Letter", and the Supporting Information files (S1 Checklist and S2 Dataset).

**Editor comment 2.** *Please amend the manuscript submission data (via Edit Submission) to include author Oswaldo E. Aguilar-Molina.*

**Response:** We thank the editor for noting this. We have amended the manuscript submission data via the Edit Submission portal to include Dr. Oswaldo E. Aguilar-Molina, who is also listed in the manuscript author list, affiliation block, and Author Contributions section (Formal analysis; Supervision). His ORCID iD will be registered through the submission system before final acceptance.

**Editor comment 3.** *If the reviewer comments include a recommendation to cite specific previously published works, please review and evaluate these publications to determine whether they are relevant and should be cited. There is no requirement to cite these works unless the editor has indicated otherwise.*

**Response:** Reviewer #1 did not request citation of any specific previously published works. Accordingly, no additional citations have been added on this basis. We have nevertheless reviewed our reference list for completeness and correctness as requested in Editor comment 4.

**Editor comment 4.** *Please review your reference list to ensure that it is complete and correct. If you have cited papers that have been retracted, please include the rationale for doing so in the manuscript text, or remove these references and replace them with relevant current references.*

**Response:** We have reviewed every reference in the reference list. None of the cited works has been retracted. All bibliographic details (authors, journal, year, volume, issue, pages) have been verified against the original sources and formatted in PLOS ONE citation style. No changes to the reference list were required.

**Response to Reviewer #1**

We are grateful to Reviewer #1 for the careful reading of our manuscript and for the encouraging overall assessment ("This study is well conducted and written"). We have addressed each of the three specific suggestions below.

**Reviewer #1, comment 1.** *In the Methods section, the author stated "The probability of pulmonary hypertension was assessed using the European Society of Cardiology/European Respiratory Society echocardiographic criteria, with TRV >2.5 m/s and >2.8 m/s used as thresholds for intermediate and high probability, respectively [18]" (page 11). However, they used a threshold of 3 m/s in the Result section (page 14) and discussion section (page 16). They should either correct this point or provide explanation to explain this change.*

**Response:** We thank the reviewer for highlighting this inconsistency, which we have now addressed. The Methods section has been expanded to explicitly state that pulmonary hypertension probability was assessed using two complementary frameworks: (i) the 2022 ESC/ERS general echocardiographic criteria, which classify TRV > 2.5 m/s and > 2.8 m/s as intermediate and high probability of pulmonary hypertension, respectively, in the absence of other echocardiographic signs [ref 18]; and (ii) the SCD-specific TRV > 3.0 m/s threshold, which has been validated in the sickle cell disease population as more specifically associated with pre-capillary pulmonary hypertension confirmed by right heart catheterisation [refs 8, 21]. We report results against both frameworks throughout the Results and Discussion to allow the reader to interpret the findings in the context of both the general pulmonary hypertension literature and the disease-specific literature. Specifically, in the Results we now report that TRV exceeded 2.5 m/s in 14 of 36 patients with measurable TRV (38.9%; 24.6% of the whole cohort) and exceeded 3.0 m/s in 6 patients (16.7% of those with measurable TRV; 10.5% of the whole cohort), with the remainder falling between 2.5 and 3.0 m/s — a range that encompasses the ESC/ERS > 2.8 m/s high-probability threshold. The Discussion now explicitly compares our findings against both the ESC/ERS framework and the SCD-specific literature. All the affected paragraphs are visible in the tracked-changes file.

**Reviewer #1, comment 2.** *Baseline clinical and laboratory characteristics should include the percentage of patients which have received blood transfusion in a delay of less than 3 months since blood transfusion may modified several biological parameters.*

**Response:** We fully agree with the reviewer that recent transfusion can modify several biological parameters (haemoglobin, haemolytic markers, NT-proBNP) and pulmonary haemodynamics (TRV), and we attempted to retrieve this information during data abstraction. Unfortunately, the date of the most recent transfusion was not consistently recorded in the source electronic medical records for this retrospective cohort and could not be ascertained reliably for all patients; the proportion of patients transfused within the three months preceding echocardiography therefore could not be reported. We have now addressed this in three places of the manuscript: (i) in the Methods (Data collection and definitions) we explicitly state that the date of the most recent blood transfusion could not be reliably ascertained from the source records; (ii) in the Results (Baseline clinical and laboratory characteristics) we now flag this issue at the level at which the reviewer requested the variable, explaining that the proportion of patients with recent transfusion could not be calculated and that this may have influenced haemoglobin, NT-proBNP and TRV; and (iii) in the Limitations section we now list incomplete transfusion history as a specific source of potential confounding that will be addressed prospectively in our planned multicentre follow-up study, in which transfusion dates are pre-specified as a mandatory data field. We thank the reviewer for the suggestion, which has substantially improved the transparency of the manuscript.

**Reviewer #1, comment 3.** *Although a limited number of patients exhibited an intermediate-to-high echocardiographic probability of pulmonary hypertension, it could be worthwhile to compare the echocardiographic characteristics of these patients to the other ones which displayed normal TRV values.*

**Response:** We agree that such a comparison is of interest and have added a dedicated descriptive paragraph to the Results section under the new sub-heading "Exploratory comparison according to tricuspid regurgitation velocity". In this paragraph we compare patients with measurable TRV > 2.5 m/s (n = 14) and those with measurable TRV ≤ 2.5 m/s (n = 22) across age, haemoglobin, left ventricular ejection fraction, left ventricular mass index, average E/e′, estimated pulmonary artery systolic pressure, indexed left and right atrial volumes, and TAPSE. The two subgroups were similar in age, haemoglobin and biventricular systolic function; as expected by design, PASP was substantially higher in the elevated-TRV group, and indexed left and right atrial volumes tended to be larger, while TAPSE was slightly lower. We have framed this as an exploratory descriptive analysis only, in line with the cross-sectional design and the modest absolute numbers, and have explicitly noted in both the new paragraph and the Limitations section that formal hypothesis testing and multivariable modelling were not pre-specified and are deferred to our planned prospective multicentre follow-up study. We thank the reviewer for this suggestion, which has added clinically meaningful texture to the manuscript without overstating the inferential strength of the data.

**Closing remarks**

We thank the Academic Editor and Reviewer #1 once again for their constructive evaluation. We believe that the changes prompted by the review process, together with the data consistency audit described at the beginning of this letter, have substantially strengthened the manuscript and the supporting dataset. We remain at your disposal for any further clarification or revision.

Yours sincerely,

**Stephany Barbosa-Balaguera, MD, MSc(c)**

On behalf of all co-authors

Corresponding author

Department of Cardiology, Universidad del Valle

Hospital Universitario del Valle "Evaristo García" E.S.E.

Cali, Colombia
