# Supplementary material for: Echocardiographic characterization and markers of cardiovascular risk in adults with sickle cell disease in a Colombian tertiary referral centre: A cross-sectional study
Source: PLoS One. 2026 Jun 16;21(6):e0348383. doi: 10.1371/journal.pone.0348383 (PMC13271432; doi:10.1371/journal.pone.0348383)
Supplement: Peer review history

# Peer review: Decision Letter 0

**DOI:** 10.1371/journal.pone.0348383.r001

**Authors:** Philippe Connes

19 May 2026

PONE-D-26-18282

Echocardiographic characterization and markers of cardiovascular risk in adults with sickle cell disease in a Colombian tertiary referral centre: a cross-sectional study

PLOS One

Dear Dr. Barbosa-Balaguera,

Thank you for submitting your manuscript to PLOS ONE. After careful consideration, we feel that it has merit but does not fully meet PLOS ONE’s publication criteria as it currently stands. Therefore, we invite you to submit a revised version of the manuscript that addresses the points raised during the review process.

Please submit your revised manuscript by Jul 03 2026 11:59PM. If you will need more time than this to complete your revisions, please reply to this message or contact the journal office at plosone@plos.org. When you're ready to submit your revision, log on to https://www.editorialmanager.com/pone/ and select the 'Submissions Needing Revision' folder to locate your manuscript file.

Please include the following items when submitting your revised manuscript:

- A letter that responds to each point raised by the academic editor and reviewer(s). You should upload this letter as a separate file labeled 'Response to Reviewers'.
- A marked-up copy of your manuscript that highlights changes made to the original version. You should upload this as a separate file labeled 'Revised Manuscript with Track Changes'.
- An unmarked version of your revised paper without tracked changes. You should upload this as a separate file labeled 'Manuscript'.

If you would like to make changes to your financial disclosure, please include your updated statement in your cover letter. Guidelines for resubmitting your figure files are available below the reviewer comments at the end of this letter.

If applicable, we recommend that you deposit your laboratory protocols in protocols.io to enhance the reproducibility of your results. Protocols.io assigns your protocol its own identifier (DOI) so that it can be cited independently in the future. For instructions see: https://journals.plos.org/plosone/s/submission-guidelines#loc-laboratory-protocols. Additionally, PLOS ONE offers an option for publishing peer-reviewed Lab Protocol articles, which describe protocols hosted on protocols.io. Read more information on sharing protocols at https://plos.org/protocols?utm_medium=editorial-email&utm_source=authorletters&utm_campaign=protocols.

As the corresponding author, your ORCID iD is verified in the submission system and will appear in the published article. PLOS supports the use of ORCID, and we encourage all coauthors to register for an ORCID iD and use it as well. Please encourage your coauthors to verify their ORCID iD within the submission system before final acceptance, as unverified ORCID iDs will not appear in the published article. *Only*   the individual author can complete the verification step; PLOS staff *cannot*   verify ORCID iDs on behalf of authors.

We look forward to receiving your revised manuscript.

Kind regards,

Philippe Connes

Academic Editor

PLOS One

**Journal Requirements:**

When submitting your revision, we need you to address these additional requirements.

1. Please ensure that your manuscript meets PLOS ONE's style requirements, including those for file naming. The PLOS ONE style templates can be found at

https://journals.plos.org/plosone/s/file?id=wjVg/PLOSOne_formatting_sample_main_body.pdf and

https://journals.plos.org/plosone/s/file?id=ba62/PLOSOne_formatting_sample_title_authors_affiliations.pdf

2. Please amend the manuscript submission data (via Edit Submission) to include author Oswaldo E. Aguilar-Molina.

3. If the reviewer comments include a recommendation to cite specific previously published works, please review and evaluate these publications to determine whether they are relevant and should be cited. There is no requirement to cite these works unless the editor has indicated otherwise.

4. Please review your reference list to ensure that it is complete and correct. If you have cited papers that have been retracted, please include the rationale for doing so in the manuscript text, or remove these references and replace them with relevant current references. Any changes to the reference list should be mentioned in the rebuttal letter that accompanies your revised manuscript. If you need to cite a retracted article, indicate the article’s retracted status in the References list and also include a citation and full reference for the retraction notice.

[Note: HTML markup is below. Please do not edit.]

Reviewers' comments:

Reviewer's Responses to Questions

**Comments to the Author**

1. Is the manuscript technically sound, and do the data support the conclusions?

The manuscript must describe a technically sound piece of scientific research with data that supports the conclusions. Experiments must have been conducted rigorously, with appropriate controls, replication, and sample sizes. The conclusions must be drawn appropriately based on the data presented.

Reviewer #1: Yes

**********

2. Has the statistical analysis been performed appropriately and rigorously?

Reviewer #1: Yes

**********

3. Have the authors made all data underlying the findings in their manuscript fully available?

The PLOS Data policy requires authors to make all data underlying the findings described in their manuscript fully available without restriction, with rare exception (please refer to the Data Availability Statement in the manuscript PDF file). The data should be provided as part of the manuscript or its supporting information, or deposited to a public repository. For example, in addition to summary statistics, the data points behind means, medians and variance measures should be available. If there are restrictions on publicly sharing data—e.g. participant privacy or use of data from a third party—those must be specified.

Reviewer #1: Yes

**********

4. Is the manuscript presented in an intelligible fashion and written in standard English?

PLOS ONE does not copyedit accepted manuscripts, so the language in submitted articles must be clear, correct, and unambiguous. Any typographical or grammatical errors should be corrected at revision, so please note any specific errors here.

Reviewer #1: Yes

**********

5. Review Comments to the Author

Please use the space provided to explain your answers to the questions above. You may also include additional comments for the author, including concerns about dual publication, research ethics, or publication ethics. (Please upload your review as an attachment if it exceeds 20,000 characters)

Reviewer #1: In the present study, the authors characterised the clinical and echocardiographic features of adults with SCD attending a tertiary referral centre in Cali, Colombia, with particular focus on structural, systolic, diastolic, valvular, and haemodynamic parameters relevant to cardiovascular risk stratification.

This study is well conduced and written.

I have few suggestions.

1. In the Methods section, the author stated “The probability of pulmonary hypertension was assessed using the European Society of Cardiology/European Respiratory Society echocardiographic criteria, with TRV >2.5 m/s and >2.8 m/s used as thresholds for intermediate and high probability, respectively [18]” (page 11). However, they used a threshold of 3 m/s in the Result section (page 14) and discussion section (page 16). They should either correct this point or provide explanation to explain this change.

2. Baseline clinical and laboratory characteristics should include the percentage of patients which have received blood transfusion in a delay of less than 3 months since blood transfusion may modified several biological parameters.

3. Although a limited number of patients exhibited an intermediate-to-high echocardiographic probability of pulmonary hypertension, it could be worthwhile to compare the echocardiographic characteristics of these patients to the other ones which displayed normal TRV values.

**********

6. PLOS authors have the option to publish the peer review history of their article (what does this mean?). If published, this will include your full peer review and any attached files.

If you choose “no”, your identity will remain anonymous but your review may still be made public.

**Do you want your identity to be public for this peer review?**   For information about this choice, including consent withdrawal, please see our Privacy Policy.

Reviewer #1: No

**********

[NOTE: If reviewer comments were submitted as an attachment file, they will be attached to this email and accessible via the submission site. Please log into your account, locate the manuscript record, and check for the action link "View Attachments". If this link does not appear, there are no attachment files.]

To ensure your figures meet our technical requirements, please review our figure guidelines: https://journals.plos.org/plosone/s/figures

You may also use PLOS’s free figure tool, NAAS, to help you prepare publication quality figures: https://journals.plos.org/plosone/s/figures#loc-tools-for-figure-preparation.

NAAS will assess whether your figures meet our technical requirements by comparing each figure against our figure specifications.

# Author comment: Author response to Decision Letter 1

**DOI:** 10.1371/journal.pone.0348383.r002

25 May 2026

Ese campo es donde PLOS ONE te pide pegar el Response to Reviewers completo dentro del portal (además de subirlo como archivo). Es el contenido del documento Word que ya te preparé, pero en texto plano para pegar directamente en la caja del portal.

Aquí te lo dejo formateado para copiar y pegar:

Manuscript: PONE-D-26-18282

Title: Echocardiographic characterization and markers of cardiovascular risk in adults with sickle cell disease in a Colombian tertiary referral centre: a cross-sectional study

Corresponding author: Stephany Barbosa-Balaguera, MD, MSc(c)

Dear Dr. Connes and Reviewer #1,

We thank you for the constructive evaluation of our manuscript and the opportunity to submit a revised version. We have carefully addressed every point raised by the Academic Editor and Reviewer #1. The accompanying revised manuscript shows all changes highlighted in yellow (no track-change marks); a comprehensive summary table of every change is also provided at the end of the Response to Reviewers file.

================================================================

PRELIMINARY NOTE — DATA CONSISTENCY AUDIT AND CORRECTIONS

Before addressing the specific comments of the Academic Editor and Reviewer #1, we wish to bring an important matter to the Editor's attention in the interest of full transparency.

In compliance with the PLOS Data Availability policy, we prepared the complete de-identified individual-level dataset (S2 Dataset) to accompany the revised manuscript. While doing so, we conducted a systematic, variable-by-variable consistency check between this dataset and the values reported in Tables 1 and 2 and in the Abstract. This audit identified several discrepancies, which we have corrected.

The discrepancies were of three kinds. First, a small number of transcription errors in individual values: median haemoglobin (corrected from 10.2 to 8.5 g/dL), median creatinine (corrected from 0.5 to 0.6 mg/dL), hydroxyurea use (corrected from 40 [71.4%] to 37 of 54 patients with available data [68.5%]), and two left ventricular geometry categories whose percentages had been inadvertently transposed (concentric remodelling, corrected to 10.5%; eccentric hypertrophy, corrected to 26.3%). Additional minor table corrections affected posterior wall thickness, relative wall thickness, and LVOT diameter. Second, several left ventricular dimensions and volumes in Table 2 required correction to match the dataset (LV end-diastolic and end-systolic diameters, LV mass index, LV end-diastolic and end-systolic volumes, stroke volume, indexed atrial volumes, and aortic root). We also identified and corrected an error in the Results text concerning indexed atrial volume: the value previously reported as "indexed left atrial volume 25.5 mL/m² (IQR 21.4–30.0), within normal limits" had inadvertently mixed the right atrial value into the left atrial sentence. The revised text now correctly reports the indexed left atrial volume as 36.1 mL/m² (IQR 25.2–42.7; n = 54), at the upper limit of normal, and the indexed right atrial volume as 25.5 mL/m² (IQR 18.1–33.0; n = 51), within normal limits. Third — and most relevant from a methodological standpoint — we identified variables with incomplete data that had been reported without an explicit denominator. Most notably, tricuspid regurgitation velocity (TRV) was measurable in 36 of the 57 patients; the proportion of patients with TRV > 2.5 m/s is now reported transparently both as a fraction of patients with measurable TRV (14 of 36; 38.9%) and of the whole cohort (24.6%), and the 21 patients without a measurable tricuspid regurgitant jet are now explicitly stated. Denominators have likewise been made explicit for NT-proBNP (n = 17), global longitudinal strain (n = 8), genotype (n = 55) and hydroxyurea use (n = 54).

We emphasise that the study population (n = 57) and all primary analyses are unchanged. These corrections affect reported values and denominators only; they do not alter the study design, the inclusion of patients, or the principal conclusions, which remain fully supported. The accompanying S2 Dataset includes a README sheet listing the available-case denominator for every variable.

================================================================

RESPONSE TO THE ACADEMIC EDITOR'S ADDITIONAL REQUIREMENTS

EDITOR COMMENT 1: Please ensure that your manuscript meets PLOS ONE's style requirements, including those for file naming.

Response: We have reviewed both PLOS ONE style templates (main body and title/authors/affiliations) and reformatted the manuscript accordingly. The title page, author list and affiliations, abstract, main body section headings, figure and table legends, and Supporting Information section now follow the PLOS ONE format. Files have been renamed in accordance with PLOS ONE conventions.

EDITOR COMMENT 2: Please amend the manuscript submission data (via Edit Submission) to include author Oswaldo E. Aguilar-Molina.

Response: We have amended the manuscript submission data via the Edit Submission portal to include Dr. Oswaldo E. Aguilar-Molina, who is also listed in the manuscript author list, affiliation block, and Author Contributions section (Formal analysis; Supervision). His ORCID iD will be registered through the submission system before final acceptance.

EDITOR COMMENT 3: If the reviewer comments include a recommendation to cite specific previously published works, please review and evaluate these publications.

Response: Reviewer #1 did not request citation of any specific previously published work. Accordingly, no additional citations have been added on this basis.

EDITOR COMMENT 4: Please review your reference list to ensure that it is complete and correct.

Response: We have reviewed every reference in the reference list. None of the cited works has been retracted. All bibliographic details (authors, journal, year, volume, issue, pages) have been verified against the original sources and formatted in PLOS ONE citation style. No changes to the reference list were required.

================================================================

RESPONSE TO REVIEWER #1

We are grateful to Reviewer #1 for the careful reading of our manuscript and for the encouraging overall assessment ("This study is well conducted and written"). We have addressed each of the three specific suggestions below.

REVIEWER #1, COMMENT 1: In the Methods section, the author stated "The probability of pulmonary hypertension was assessed using the European Society of Cardiology/European Respiratory Society echocardiographic criteria, with TRV >2.5 m/s and >2.8 m/s used as thresholds for intermediate and high probability, respectively [18]" (page 11). However, they used a threshold of 3 m/s in the Result section (page 14) and discussion section (page 16). They should either correct this point or provide explanation to explain this change.

Response: We thank the reviewer for highlighting this inconsistency, which we have now addressed. The Methods section has been expanded to explicitly state that pulmonary hypertension probability was assessed using two complementary frameworks: (i) the 2022 ESC/ERS general echocardiographic criteria, which classify TRV > 2.5 m/s and > 2.8 m/s as intermediate and high probability of pulmonary hypertension, respectively, in the absence of other echocardiographic signs [ref 18]; and (ii) the SCD-specific TRV > 3.0 m/s threshold, which has been validated in the sickle cell disease population as more specifically associated with pre-capillary pulmonary hypertension confirmed by right heart catheterisation [refs 8, 21]. We report results against both frameworks throughout the Results and Discussion to allow the reader to interpret the findings in the context of both the general pulmonary hypertension literature and the disease-specific literature. Specifically, in the Results we now report that TRV exceeded 2.5 m/s in 14 of 36 patients with measurable TRV (38.9%; 24.6% of the whole cohort) and exceeded 3.0 m/s in 6 patients (16.7% of those with measurable TRV; 10.5% of the whole cohort), with the remainder falling between 2.5 and 3.0 m/s — a range that encompasses the ESC/ERS > 2.8 m/s high-probability threshold. The Discussion now explicitly compares our findings against both the ESC/ERS framework and the SCD-specific literature. All the affected paragraphs are highlighted in yellow in the revised manuscript.

REVIEWER #1, COMMENT 2: Baseline clinical and laboratory characteristics should include the percentage of patients which have received blood transfusion in a delay of less than 3 months since blood transfusion may modified several biological parameters.

Response: We fully agree with the reviewer that recent transfusion can modify several biological parameters (haemoglobin, haemolytic markers, NT-proBNP) and pulmonary haemodynamics (TRV), and we attempted to retrieve this information during data abstraction. Unfortunately, the date of the most recent transfusion was not consistently recorded in the source electronic medical records for this retrospective cohort and could not be ascertained reliably for all patients; the proportion of patients transfused within the three months preceding echocardiography therefore could not be reported. We have now addressed this in three places of the manuscript: (i) in the Methods (Data collection and definitions) we explicitly state that the date of the most recent blood transfusion could not be reliably ascertained from the source records; (ii) in the Results (Baseline clinical and laboratory characteristics) we now flag this issue at the level at which the reviewer requested the variable, explaining that the proportion of patients with recent transfusion could not be calculated and that this may have influenced haemoglobin, NT-proBNP and TRV; and (iii) in the Limitations section we now list incomplete transfusion history as a specific source of potential confounding that will be addressed prospectively in our planned multicentre follow-up study, in which transfusion dates are pre-specified as a mandatory data field. We thank the reviewer for the suggestion, which has substantially improved the transparency of the manuscript.

REVIEWER #1, COMMENT 3: Although a limited number of patients exhibited an intermediate-to-high echocardiographic probability of pulmonary hypertension, it could be worthwhile to compare the echocardiographic characteristics of these patients to the other ones which displayed normal TRV values.

Response: We agree that such a comparison is of interest and have added a dedicated descriptive paragraph to the Results section under the new sub-heading "Exploratory comparison according to tricuspid regurgitation velocity". In this paragraph we compare patients with measurable TRV > 2.5 m/s (n = 14) and those with measurable TRV ≤ 2.5 m/s (n = 22) across age, haemoglobin, left ventricular ejection fraction, left ventricular mass index, average E/e′, estimated pulmonary artery systolic pressure, indexed left and right atrial volumes, and TAPSE. The two subgroups were similar in age, haemoglobin and biventricular systolic function; as expected by design, PASP was substantially higher in the elevated-TRV group, and indexed left and right atrial volumes tended to be larger, while TAPSE was slightly lower. We have framed this as an exploratory descriptive analysis only, in line with the cross-sectional design and the modest absolute numbers, and have explicitly noted in both the new paragraph and the Limitations section that formal hypothesis testing and multivariable modelling were not pre-specified and are deferred to our planned prospective multicentre follow-up study.

================================================================

CLOSING REMARKS

We thank the Academic Editor and Reviewer #1 once again for their constructive evaluation. We believe that the changes prompted by the review process, together with the data consistency audit described at the beginning of this letter, have substantially strengthened the manuscript and the supporting dataset. The full point-by-point response with a comprehensive summary table of every change made is also provided as a separate "Response to Reviewers" file uploaded with this revision. We remain at your disposal for any further clarification or revision.

Yours sincerely,

Stephany Barbosa-Balaguera, MD, MSc(c)

On behalf of all co-authors

Attachment Submitted filename: Response_to_Reviewers_4.docx

# Editor report: Decision Letter 1

**DOI:** 10.1371/journal.pone.0348383.r003

**Authors:** Philippe Connes

1 Jun 2026

Echocardiographic characterization and markers of cardiovascular risk in adults with sickle cell disease in a Colombian tertiary referral centre: a cross-sectional study

PONE-D-26-18282R1

Dear Dr. Barbosa-Balaguera,

We’re pleased to inform you that your manuscript has been judged scientifically suitable for publication and will be formally accepted for publication once it meets all outstanding technical requirements.

Within one week, you’ll receive an e-mail detailing the required amendments. When these have been addressed, you’ll receive a formal acceptance letter and your manuscript will be scheduled for publication.

An invoice will be generated when your article is formally accepted. Please note, if your institution has a publishing partnership with PLOS and your article meets the relevant criteria, all or part of your publication costs will be covered. Please make sure your user information is up-to-date by logging into Editorial Manager at Editorial Manager® and clicking the ‘Update My Information' link at the top of the page. For questions related to billing, please contact billing support.

If your institution or institutions have a press office, please notify them about your upcoming paper to help maximize its impact. If they’ll be preparing press materials, please inform our press team as soon as possible -- no later than 48 hours after receiving the formal acceptance. Your manuscript will remain under strict press embargo until 2 pm Eastern Time on the date of publication. For more information, please contact onepress@plos.org.

Kind regards,

Philippe Connes

Academic Editor

PLOS One

Additional Editor Comments (optional):

Reviewers' comments:

# Editor report: Acceptance letter

**DOI:** 10.1371/journal.pone.0348383.r004

**Authors:** Philippe Connes

PONE-D-26-18282R1

PLOS One

Dear Dr. Barbosa-Balaguera,

I'm pleased to inform you that your manuscript has been deemed suitable for publication in PLOS One. Congratulations! Your manuscript is now being handed over to our production team.

At this stage, our production department will prepare your paper for publication. This includes ensuring the following:

* All references, tables, and figures are properly cited

* All relevant supporting information is included in the manuscript submission,

* There are no issues that prevent the paper from being properly typeset

You will receive further instructions from the production team, including instructions on how to review your proof when it is ready. Please keep in mind that we are working through a large volume of accepted articles, so please give us a few days to review your paper and let you know the next and final steps.

Lastly, if your institution or institutions have a press office, please let them know about your upcoming paper now to help maximize its impact. If they'll be preparing press materials, please inform our press team within the next 48 hours. Your manuscript will remain under strict press embargo until 2 pm Eastern Time on the date of publication. For more information, please contact onepress@plos.org.

You will receive an invoice from PLOS for your publication fee after your manuscript has reached the completed accept phase. If you receive an email requesting payment before acceptance or for any other service, this may be a phishing scheme. Learn how to identify phishing emails and protect your accounts at https://explore.plos.org/phishing.

If we can help with anything else, please email us at customercare@plos.org.

Thank you for submitting your work to PLOS ONE and supporting open access.

Kind regards,

PLOS ONE Editorial Office Staff

on behalf of

Pr Philippe Connes

Academic Editor

PLOS One
